# Successful Detection of the Characteristics of Tear Film Breakup Appearing Immediately after Eye Opening by Videokeratography with a Newly-Developed Indicator

**DOI:** 10.3390/diagnostics13020240

**Published:** 2023-01-09

**Authors:** Norihiko Yokoi, Natsuki Kusada, Hiroaki Kato, Yuki Furusawa, Chie Sotozono, Georgi As. Georgiev

**Affiliations:** 1 Department of Ophthalmology, Kyoto Prefectural University of Medicine, Kyoto 602-8566, Japan; 2 Faculty of Physics, Sofia University “St. Kliment Ohridski”, 1164 Sofia, Bulgaria

**Keywords:** decreased wettability dry eye, disturbance value, spot break, tear film breakup pattern, videokeratography

## Abstract

Spot break (SB), a tear film breakup (TFBU) subtype seen in decreased wettability dry eye (DE), is characterized by a spot-like TFBU that appears immediately after eye opening. It is sometimes difficult to detect using currently available devices for evaluating non-invasive TFBU. The purpose of this study was to investigate the effectiveness of using a newly developed videokeratography indicator for detecting SB. The study involved 44 eyes of 44 DE patients (21 eyes with SB (SB group) and 23 eyes with random break in which fluorescein breakup time was ≤ 5 s (s) (RB group)). All eyes were examined using videokeratography, with digital Meyer-ring images being obtained. By calculation of the degree of luminance blur on the cornea in the Meyer-ring images, termed ‘disturbance value’ (DV), DVs at 0 s (DV(0)]), 2 s (DV(2)), and 5 s (DV(5)) after eye opening, and the changes of DV between each time, were compared between the SB and RB groups. Results: No significant differences in DV(2) and DV(5) and the rate of change between DV(2) and DV(5) were found between the two groups. However, DV(0) and rate of change between DV(0) and DV(2) in the SB group were significantly greater (*p* < 0.001) than those in the RB group. SB characteristics were successfully detected by videokeratography using a new videokeratography DV indicator.

## 1. Introduction

Dry eye (DE), a common ocular surface (OS) disorder, is on the rise globally due to an increase in elderly populations and the greater use of visual display terminals and contact lenses, as well as other factors [1]. It has also been reported that DE results in economic loss by reduced work efficiency due to DE-related symptoms such as ocular discomfort and/or visual impairment [2].

For clinical screening and assessment of the severity of DE, evaluation of fluorescein staining of the OS epithelial damage, and of fluorescein breakup time (FBUT) of the tear film (TF), are most commonly used, since an unstable TF with a breakup time of ≤5 s plays a central role in the disease [3,4]. However, the advancement of several non-invasive methods for evaluating the OS has been reported, including videointerferometry (VI) [5,6,7,8], videothermography [9], wavefront aberrometry [10,11,12,13], and videokeratography (VK) [14,15]. These non-invasive methods have the advantages of being less influenced by the examiner’s personal technique and skills, and result in minimal reflex tearing and more reliable estimates of TF stability in comparison with the invasive fluorescein-based examinations [1,3,4]. Therefore, the Dry Eye Workshop of the Tear Film & Ocular Surface Society Eye (TFOS DEWS II) also recommends evaluating non-invasive breakup time (NIBUT) of the TF as one of the criteria used for the diagnosis of DE [4].

However, at the moment, non-invasive methods for evaluating the OS have several shortcomings that hamper their broad clinical adoption, such as a limited field of observation over the cornea, no system that can be used for tracking eye motion, and limited quantification functionality. These shortcomings can result in a limited assessment of specific corneal-surface abnormalities that are expressed in a variety of DE subtypes.

As summarized by the Asia Dry Eye Society (ADES) consensus report [16], DE is a multifactorial disease with three subtypes: (1) aqueous deficient DE (ADDE; (2) increased evaporation DE (IEDE), and (3) decreased wettability DE (DWDE). Each of the DE subtypes can be differentially diagnosed based on the fluorescein breakup time (FBUT) and the characteristic spatial fluorescein breakup pattern (FBUPs) across the ocular surface. Therefore, ADES has now embraced ‘Tear Film Oriented Diagnosis’ (TFOD) and ‘Tear Film Oriented Therapy’ (TFOT), two new concepts developed in Japan, for the diagnosis and therapy of DE [5,16,17,18,19,20]. In TFOD, there are five essential FBUPs including: area break (AB) and line break (LB); spot break (SB) and dimple break (DB), and random break (RB), suggesting the existence of ADDE, DWDE, and (iii) IEDE, respectively [5,16,17,18,19,20]. When TFOD and TFOT are applied, insufficient components of the OS responsible for TF breakup are elucidated, with DE then selectively treated by the improvement of TF stability by supplementation of insufficient components to the patient’s ocular surface.

To the best of our knowledge, no comprehensive and non-invasive method for the evaluation of DE based on TF breakup patterns (BUPs) has been reported. Such a method would be of great benefit, as BUPs, irrespective of different corneal surface manifestations, can, via the application of TFOD and TFOT, identify each DE subtype and the respective optimal therapy for each patient’s DE. Moreover, when quantitative detection of BUPs eventually becomes possible via a non-invasive method, it may lead to not only ophthalmologists, but also other medical staff members, applying the TFOD pathway, and further lead to the development of new artificial-intelligence-supported diagnostic methods.

Currently, videokeratography is considered to have advantages over the other non-invasive methods used for the evaluation of DE, as it covers a greater corneal area for the assessment. In addition, VK produces images of better contrast, including brighter Meyer-ring (MR) images and black-background images of the other part of the cornea, thus allowing better quantitative assessment of dynamic changes of the corneal surface abnormalities in DE.

The purpose of the present study was to investigate whether a newly-developed videokeratography software indicator can be used to quantitatively detect and discriminate between some DE breakup patterns. The algorithm analyzes blur value (BV) and disturbance value (DV), i.e., indicators that exhibit the extent of the reflection of placid rings of the VK instrument at the precorneal TF surface. These indicators correspond to the disturbance of Meyer-ring (MR) images and aim to quantitatively summarize the spatiotemporal evolution of the irregularity of the precorneal TF and/or corneal surface epithelium as seen in cases of DE. Random break and spot break were chosen as test BUPs. RB served as a control as it is the pattern most commonly observed in evaporation-related TF instability both in IEDE and in healthy eyes [5,16,17,18,19,20]. In contrast SB, which appears immediately after the eye is opened, is indicative of the so-called short breakup time DE [21], a clinically important DWDE subtype. Despite the very short BUT and severe DE symptoms, SB represents a diagnostic challenge and is often overlooked. This is due to SB confinement over a small corneal spot, there is minimal or no corneal epithelial damage, and it has been reported to be the most difficult BUP to detect via the use of a wavefront analyzer [13]. Thus, a reliable videokeratography identification of SB will provide a valuable tool for differential diagnoses of dry eye.

## 2. Materials and Methods

The protocols of this cross-sectional comparative study were approved by the Institutional Review Board of Kyoto Prefectural University of Medicine, Kyoto, Japan (Approval No. ERB-C-1233-4). The study was conducted in accordance with the tenets set forth in the Declaration of Helsinki, and written informed consent was obtained from all patients prior to their involvement in the study.

### 2.1. Subjects

The study involved 44 eyes (15 right eyes and 29 left eyes) of 44 DE patients (4 males and 40 females; mean age: 66.1 ± 10.7 (mean ± SD) years) seen post referral at the Department of Ophthalmology Dry Eye Clinic of Kyoto Prefectural University of Medicine between January 2020 and December 2021. All patients were diagnosed with DE based on the current Japanese diagnostic criteria [22]; i.e., exhibiting the DE-related symptoms of eye discomfort and/or visual impairment, and a fluorescein breakup time (FBUT) of ≤5 s. The eyes deemed eligible for involvement in this study were eyes with more severe symptoms. In cases in which the severity of the symptoms was identical in both eyes, the right-eye data were used. Prior to enrollment in the study, confirmation was obtained from all patients that no eye-drop medications had been used for at least 1 h before the initial examination.

All subjects with an eyelid disease such as blepharoptosis, lagophthalmos, blepharospasm, entropion, or ectropion, as well as those with severe conjunctivochalasis or any history of eye surgery, including those for the puncta, OS diseases, the eyelid, and glaucoma, were excluded from the study. In addition, ADDE patients were excluded from the study even when SB was observed, as ADDE can accompany SB, thus implying decreased corneal wettability that may be caused secondarily via the shedding of membrane-associated mucins due to the OS inflammation associated with ADDE [23,24]. Moreover, all patients deemed inappropriate for involvement in this study based on the above-described reasons, or other reasons, were excluded via consensus by three ophthalmologists (N.Y., H.K., N.K.) following a review of the data.

### 2.2. Study Protocol

On the day of examination, FBUT and FBUPs were assessed by the following steps. First, two drops of saline were placed onto a fluorescein test strip (Ayumi Pharmaceutical Corporation, Tokyo, Japan) that was vigorously shaken to minimize, as much as possible, the amount of saline on the strip. Using this procedure, no significant difference of meniscus height, implying no significant tear volume increase, was noted between with fluorescein staining and without fluorescein staining (unpublished data). Next, the test strip was touched to the center of the patient’s lower lid margin, with the patient then verbally instructed to blink several times to mix the fluorescein with the aqueous tears. The patient was then instructed to briskly open the eye three consecutive times after gently closing the eye, followed by keeping the eye open as long as 5 s each time the eye was opened. Those procedures were observed by slit-lamp microscopy using appropriate filters for observing fluorescein, and were video-recorded, during which time FBUT and FBUP, respectively, were measured and classified three times, with the associated corneal epithelial damage score (CEDS) then evaluated based on the NEI Grading System [25]. The eyes that repeatedly presented an SB or RB at each of the three successive blinks were enrolled in this study, and then respectively categorized into either the SB group or RB group. Originally, RB was characterized as an FBUP in which the breakup appears after the upward movement of fluorescein has stopped (i.e., corresponding to complete establishment of the precorneal TF). In this present study, the eyes in which the fluorescein breakup appeared at 5 s after the eye was kept open were categorized as RB based on the Japanese criteria for DE [22].

In both the SB and RB groups, tear volume over the OS was evaluated via videomeniscometry (VM) at more than 15 min after the confirmation of the reproducibility of the FBUPs [26,27] and the spread grade (SG) of the precorneal TF lipid layer (TFLL), and the NIBUT were assessed using a videointerferometry instrument (DR-1^®^; Kowa Co., Ltd., Nagoya, Japan) [6,18]. Finally, the time-dependent change of precorneal TF behavior was assessed using a videokeratography instrument (RET-700; Rexxam Co., Ltd., Osaka, Japan) in which the newly-developed software indicator was incorporated.

### 2.3. Clinical Assessment

#### 2.3.1. Tear Volume over the OS

In all patients, videomeniscometry was used to measure the tear meniscus (TM) radius (TMR, mm) at the central lower TM as an index of the total aqueous tear volume over the OS. In this method, the line width in the reflected image at the TM given by a VM instrument equipped with the illuminated target with horizontal stripes was used to calculate the TMR using the previously reported concave mirror formula [26]. The TMR reflects not only the tear volume at the TM and the aqueous TF thickness of the precorneal TF, but also the total tear volume over the OS [27].

#### 2.3.2. Precorneal TFLL Spread Grades and the Measurement of NIBUT

First, using a videointerferometry instrument (DR-1^®^), the TFLL SG (i.e., a spread grade of 1–5, with 1 being the best) was evaluated. This grading system is based on the behavior of the upward spread of the TFLL (i.e., the speed, and to what extent, the TFLL covers the underlying aqueous layer) being classified into 1 of the following 5 grades: Grade 1: quick and complete spreading; Grade 2: slow and complete spreading; Grade 3: slow and partial spreading (i.e., a spread of >50% of the observed area); Grade 4: slow and partial spreading (i.e., a spread of ≤50% of the observed area); Grade 5: no spreading [18]. There is a significant relationship between the behavior of the upward spread of the TFLL and the TMR [8,18], with a greater grade reflecting a lesser tear volume over the OS [8]. Following assessment of the SG, the NIBUT was measured. In order to avoid reflex tear secretion, NIBUT was measured once up to 10 s, and NIBUT was determined at 10 s, when no breakup appeared after keeping the eye open for 10 s, and without keeping the eye open more than 10 s, and NIBUT was determined as 0 s, when breakup appeared immediately after eye opening and did not disappear even after the stoppage of upward spread of TFLL (corresponding to SB case in this study) [20].

#### 2.3.3. Assessment of VK Blur Value (BV) and Disturbance Value (DV)

**Figure 1:**
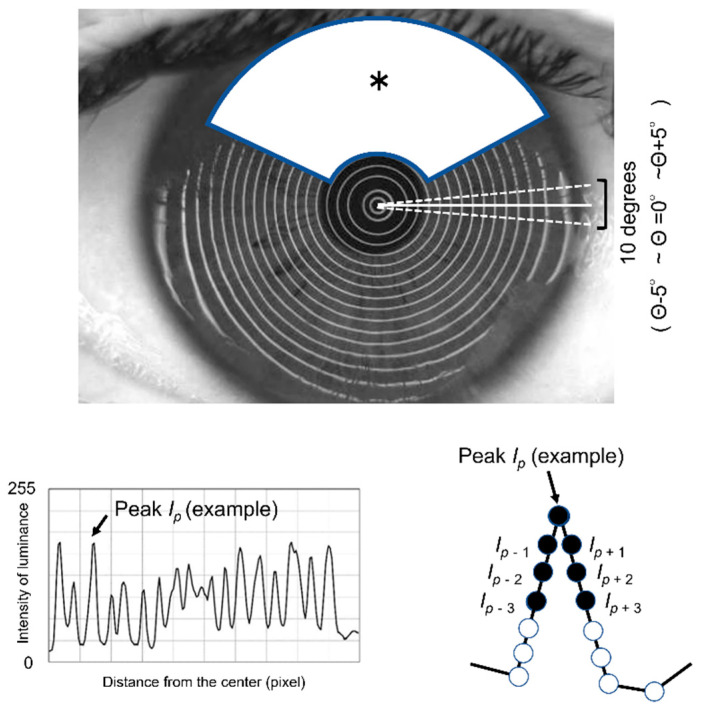
Images outlining the first step used for determining the blur value (BV) and disturbance value (DV) using a videokeratography (VK) instrument. Briefly, while tracking the center of the Meyer-ring (MR) image, the meridian extending from the center to the periphery of the cornea was determined for every 1° within a 10° (θ − 5° ~ θ = 0° ~ θ + 5°) unit (Figure 1, **upper** photo). Next, a graph indicating the relationship between the distance (pixel) from the center and the intensity of the luminance (0 ~ 255) was made (lower left image, a representative graph profile of the meridian at θ = 0° shown in the **upper** photo). From each intensity of luminance profile along each meridian within the 10° unit, the BV (an arbitrary unit) was then calculated using the formula described in the text after selecting the intensity of luminance at and around the peak (representative examples are shown in the lower left and right images). Due to the influence of the upper eyelid and eye lashes, an area from 30° to 150° external to the 5th ring (white area (*) in **upper** photo) were excluded from the analysis.

**Figure 2:**
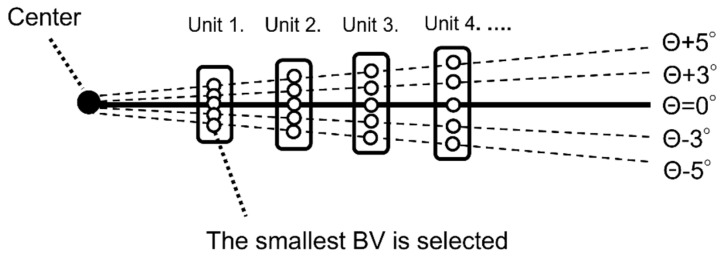
Diagram illustrating the second step used for determining the BV and DV via VK. Briefly, after completing the calculation of the BV for the peak along each meridian within every 10° unit, the smallest BV within the unit was designated as the representative BV for each unit, and the BV of the whole corneal region was then analyzed. For simplification, note that only units 1~4 are shown and that the meridians for Θ + 1, Θ + 2, Θ + 4, Θ − 1, Θ − 2, and Θ − 4 are omitted, with the associated plot within each 1–4 unit representing the calculated BV. It is also important to note that due to the influence of the upper eyelid and eye lashes on the images, an area from 30° to 150° external to the 5th ring (white area (*) in Figure 1) was excluded from the analysis. The DV (arbitrary units) was then determined as the sum of the BVs outside the excluded region.

Blur value (BV) and disturbance value (DV) are videokeratography indicators that show the extent to which BV corresponds to the reflection of placid rings of the VK instrument at the precorneal TF surface. to the disturbance of Meyer-ring (MR) images, and BV and DV are used to objectively evaluate the corneal surface irregularity comprising that of the precorneal TF and/or corneal surface epithelium seen in cases of DE, as shown in Figure 1 and Figure 2. Briefly, VK MR images were recorded while the patient’s eyes were kept open for at least 5 s after being briskly opened [18]. The images were then recorded for 15 s (10 frames per second), during which period blinks were permitted, which are useful for confirming the reproducibility of the time-dependent profile of the DV after eye opening, with a total of 150 images obtained. The DV was then calculated from all images recorded on the video using the following steps. First, while tracking the center of the MR, the meridian extending from the center to the periphery of the cornea was determined for every 1° within a 10° (θ − 5° ~ θ = 0° ~ θ + 5°) unit (Figure 1, upper photo). Next, a graph indicating the relationship between the distance (pixel) from the center and the intensity of the luminance (0 ~ 255) was made (Figure 1, lower left image (a representative graph profile of the meridian at θ = 0° shown in the upper photo)).

From each intensity of luminance profile along each meridian within the 10° unit, the BV (with arbitrary units) was calculated using the following formula after selecting the intensity of luminance at and around the peak (representative example images are shown in the lower left and right areas of Figure 1):

(1) $$BV=b-a\times \overset{k}{\underset{m=-k}{\sum }}\;\left|I_{p}-I_{p+m}\right|$$

In Equation (1), “*I_p_*” indicates the luminance value of the pixel at the peak (Figure 1, lower left and right), and “*I_p+m_*” indicates the luminance value of the pixels neighboring to the peak pixel, while “*a*”, “*b*” and “*k*” are constants related to the detection sensitivity (*a* = 4, *b* = 300, and *k* = 3, respectively).

Briefly, after completing the calculation of the BV for the peak along each meridian within every 10° unit, the smallest BV within the unit was designated as the representative BV for each unit, and the BV of the whole corneal region was then analyzed (Figure 2). For simplification, note that in Figure 2, only units 1–4 are shown, and that the meridians for Θ + 1, Θ + 2, Θ + 4, Θ − 1, Θ − 2, and Θ − 4 are omitted, with the associated plot within each 1~4 unit representing the calculated BV. It is also important to note that due to the influence of the upper eyelid and eye lashes on the images, an area from 30° to 150° external to the 5th ring (white area (*) in Figure 1) was excluded from the analysis. The DV (arbitrary unit) was then determined as the sum of the BVs outside the excluded region, with the DV calculated for each image and the time-dependent change of DV presented by a graph.

Among the images included in the analysis, those that were regarded as inappropriate for the evaluation of DV due to eye movements or other factors were excluded from the analysis.

#### 2.3.4. Analytical Parameters Related to DV

**Figure 3:**
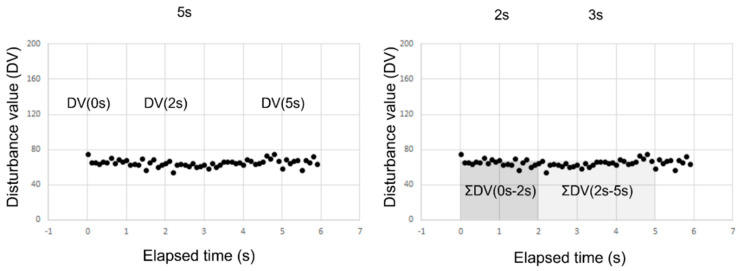
Parameters related to DV. The VK instrument provides a graph presenting time-dependent change of DVs from the time point immediately after eye opening (i.e., time = 0 s (s)). Seven parameters associated with the DVs were then obtained from the graph, including DV immediately after eye opening (DV(0 s)) and at 2 s and 5 s after eye opening (DV(2 s) and DV(5 s), respectively) (shown in the left graph), the rate of change of DV from 0 s to 2 s and from 2 s to 5 s after eye opening, defined by the equations (DV(2 s) − DV(0 s))/2 and (DV(5 s) − DV(2 s))/3, respectively, and the sum of DV from 0 s to 2 s and from 2 s to 5 s after eye opening, ΣDV(0 s–2 s) and ΣDV(2 s–5 s), respectively (shown in the right graph). DV was calculated every 0.1 s.

As shown in Figure 3, in all cases in group SB and group RB, DV immediately after eye opening (DV(0 s)), DV at 2 s after eye opening (DV(2 s)), DV at 5 s after eye opening D(V(5 s)), the rate of DV change between 0 s and 2 s (ΔDV(0 s–2 s)), the rate of DV change between 2 s and 5 s (DV(2 s–5 s)), and the sum of the DV between 0 s and 2 s (ΣDV(0 s–2 s)) and between 2 s and 5 s (ΣDV(2 s–5 s)) were calculated, and each parameter was then compared between the two groups.

In the above-described calculation, ΔDV(0 s–2 s) and ΔDV(0 s–5 s), respectively, were then calculated via Equations (2) and (3):

ΔDV(0 s–2 s) = [DV(2 s) − DV(0 s)]/2(2)

ΔDV(2 s–5 s) = [DV(5 s) − DV(2 s)]/3(3)

### 2.4. Statistical Analyses

The Mann-Whitney U test was used for comparison of TMR, SG, NIBUT, and parameters for evaluating the DV between the SB and RB groups. Statistical analyses were performed using JMP Pro version 15.0 statistics software (SAS Institute Inc., Cary, NC, USA) for the Microsoft Windows 10 operating system. A *p*-value of <0.05 was considered statistically significant.

## 3. Results

### 3.1. Demographic Data and Clinical Characteristics of the Patients

This study involved 44 patients diagnosed with DE based on the Japanese diagnostic criteria [22], and included 21 “spot break” eyes (SB group; 1 male and 20 females, mean age: 65.3 ± 8.50 (mean ± SD) years)and 23 “random break” eyes being observed within 5 s after eye opening (RB group; 3 males and 20 females, mean age: 66.9 ± 12.5 years) (Table 1). There were no significant differences in gender ratio (*p* = 0.609) or age (*p* = 0.404) between the two groups.

The mean FBUT in the SB group was 0 s ± 0 s (mean ± SD), as the SBs appeared immediately after eye opening. In contrast, the mean FBUT in the RB group was 3.83 s ± 0.93 s (range: 1.67 s ~ 5.00 s), thus showing a significant difference in mean FBUT between the two groups. The mean corneal staining scores as evaluated by use of the NEI Grading System in the SB and RB groups were 0.62 ± 0.86 (mean ± SD) and 0.26 ± 0.69, respectively, with no significant difference found between the two groups (*p* = 0.071).

### 3.2. Comparison of Tear Meniscus Radius between the SB and RB Groups

In the SB and RB groups, the mean TMR values were 0.188 ± 0.080 (mean ± SD) and 0.221 ± 0.082, respectively, with no significant difference found between the two groups (*p* = 0.346) (Table 2).

### 3.3. Comparison of TFLL Spread Grades between the SB and RB Groups

In the SB and RB groups, the mean SG values were 1.48 ± 0.51 (mean ± SD) and 1.39 ± 0.58, respectively, with no significant difference found between the two groups (*p* = 0.485) (Table 2).

### 3.4. Comparison of the Disturbance Value Parameters between the SB and RB Groups

The DV parameters (calculated values (mean ± SD) for the SB group; calculated values (mean ± SD) for the RB group) were (DV(0 s): 77.2 ± 46.6; 36.6 ± 21.8), (DV(2 s): 37.1 ± 29.9; 27.5 ± 14.7), [(V(5 s): 38.2 ± 30.1; 28.6 ± 14.3), (ΔDV(0 s–2 s): −20.0 ± 14.4; −4.56 ± 8.78), (ΔDV(2 s–5 s): 0.38 ± 3.72; 0.36 ± 2.55), (ΣDV(0 s–2 s): 953 ± 657; 570 ± 290), and (ΣDV(2 s–5 s): 1050 ± 774; 810 ± 425), (Table 3).

In general, in the RB group, DV reflecting the irregularity of the MR image looked stable for more than 5 s after the eye was opened, without any apparent DV change during that time period. In contrast, in the SB group, DV appeared greatest immediately after eye opening (i.e., within a few seconds), followed by a lessening of the DV leading to the baseline with relatively constant DV values compatible with those in the RB group.

**Figure 4:**
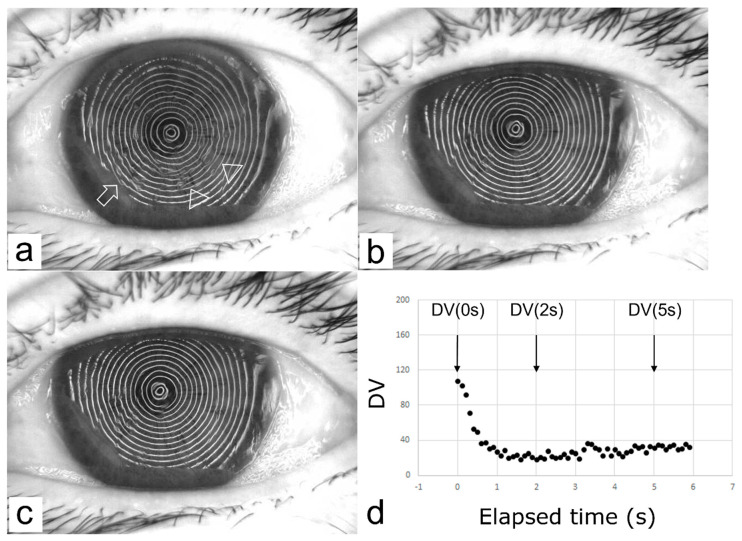
A representative case in the Spot Break (SB) group (the left eye of a 77-year-old female). MR distortions located at the temporal and lower part of the cornea appeared immediately after eye opening ((**a**), arrowheads) were found to have already disappeared at 2 s (**b**) after the eye was kept open, a condition that remained even at 5 s (**c**) after eye opening. In contrast, MR distortion at the nasal lower part of the cornea appeared immediately after eye opening ((**a**), arrow) remained unchanged during 5 s eye opening. A graph (**d**) represents this dramatic disappearance and consistent appearance of MR distortions by the disturbance value (DV) change over time. The stable baseline value of DV is thought to reflect that from the MR distortion at the nasal lower part.

**Figure 5:**
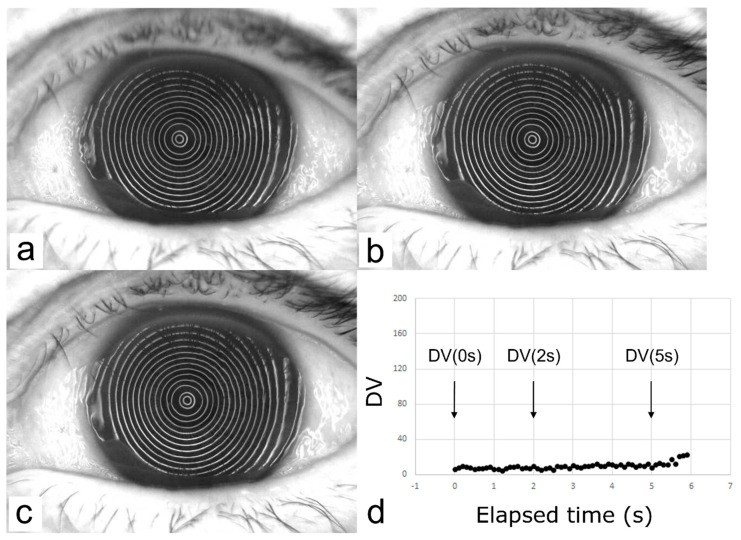
A representative case in the Random Break (RB) group (the left eye of an 81-year-old female). No MR distortion was seen at any area of the cornea or in any images ((**a**), immediately after eye opening; (**b**,**c**), 2 s and 5 s after keeping the eye open, respectively) that are reflected on the graph; thus showing almost constant DV over time (**d**).

Time-dependent changes in the VK Meyer-ring images and the corresponding DV changes in the representative SB group and RB group cases are shown in Figure 4 and Figure 5, respectively, and the results of the OS examinations in those cases are shown in Table 3. Representative movies which show SB (Videos S1 and S2) and RB (Videos S3 and S4) visualized with fluorescein staining and via VK are provided as supplementary files.

## 4. Discussion

Spot break, one of the essential DE breakup patterns indicating DWDE, was first introduced by our group in 2013 [17]. It is characterized by the sudden appearance of spot-like TFBUs immediately after eye opening prior to TFLL spread taking place [20]. When TFLL spread grade and aqueous tear amount, as reported by TMR, are normal, such behavior clearly suggests that SB is caused by locally impaired wettability of the corneal surface at the breakup spot region, in agreement with the theoretical predictions that TF breakup can be initiated even by a mildly non-wettable corneal speck with the size of a single superficial squamous cell (i.e., ~20–40 µm in diameter) [28,29]. The most probable reason for the occurrence of such a discrete, mildly non-wettable corneal speck is thought to be deficiency/impairment of MUC16 [20,30]. Such MUC-16 deficient spots become prone to lipid contamination, which result in water repellent dewetting SB upon eye opening [29]. When lipid contamination at this region is insufficient, lipids may be detached from it by the aqueous tears dragged upwards by the TFLL spread [31,32]. Thus, some the SBs are temporary and disappear within 2–3 s after eye opening, while other SBs are durable and remain uncovered by the tear fluid. In the latter case, the TFLL spreading and the aqueous tear front circumvent the spot breakup. The therapy for SB involves the use of diquafosol sodium or rebamipide eye-drops which gradually recover the SB pattern to normal after months of treatment, closely matching the anticipated course of the MUC16 recovering action of the drugs [33,34,35].

As mentioned above, the time-dependent dynamic behavior of the TF in an SB is quite unique in comparison to that in other BUPs, thus making an SB difficult to evaluate non-invasively and quantitatively. According to the findings in a report by Koh et al., a SB cannot be detected by wavefront analysis [11,12] due to its rapid and dynamic behavior [13], which appears immediately at eye opening with some subsequently disappearing at TFLL spread. However, the findings in our study demonstrated that our new videokeratography system, which utilizes a tracking system and disturbance value, and a new indicator to quantitatively assess the degree of disturbance of the MR image (i.e., reflected images of placido rings projected onto the cornea via the VK system), successfully detected the time-dependent dynamic changes of the irregularity of the TF surface associated with SB. As is shown in Figure 4, the DV in the SB group showed greater values immediately after eye opening, which then decreased to values equivalent to those in the RB group from 2 s to 5 s, with the rate of change from 0 s to 2 s greatly differing between the two groups. In contrast, and as shown in Figure 5, the DV was stable over time, showing lower values from 0 s to 5 s in the RB group, and there were no significant differences found between the SB and RB groups in regard to DV(2 s), DV(5 s), ΔDV(2 s–5 s), and ΣDV(2 s–5 s), which reflects a time-dependent profile of the TF surface from 2 s to 5 s after eye opening. Thus, from the aspect of TF dynamics, the dynamic change of the TF surface in an SB is rapid, yet transient, which occurs within 2 s after eye opening, i.e., the dynamic change observed in a SB has already subsided at 2 s after eye opening, and is compatible with that of a RB from 2 s to 5 s after eye opening.

Since no significant differences in spread grade, corneal epithelial damage score, and tear meniscus radius were found between the SB and RB groups in this study, we deemed the aqueous tear film thickness, corneal epithelial integrity, and aqueous tear volume over the OS to be similar between the two groups. Thus, VK using the tracking system and DV revealed that the immediate and greater change of the surface irregularity in SB was the only difference between the SB and RB groups. In addition, and although no significant difference was found (*p* = 0.06), the finding that ΣDV(0 s–2 s) in the SB group tended to be greater than that in the RB group may reflect the likelihood of the presence of a greater disturbance in the MR images in the SB group. Therefore, the findings conclusively suggest that the greater DV at time 0 s, followed by the dramatic decrease of DV leading to the baseline at around 2 s, must be a characteristic profile for DV in an SB.

In our clinical experience, an SB is sometimes seen together with an LB in ADDE cases, and an SB seen in ADDE is also thought to be associated with decreased corneal surface wettability. This is because in ADDE, inflammatory mediators are likely to be accumulated in tears as a result of tear deficiency-associated delayed tear clearance [36], which may cause a shedding of MUC16 that leads to decreased wettability due to the resultant dysfunction of MUC16 [23,24,35]. In fact, it is interesting that when ADDE cases presenting LB and SB together are evaluated using our VK system, the DV profile characteristic of SB demonstrated in this study were also observed. Thus, the DV profile characteristic of SB alone is not enough to determine whether a patient’s DE is an aqueous-deficient or dewetting dry eye. However, SB in DWDE and in ADDE can be differentiated by baseline disturbance values, as those tend to be greater in ADDE reflecting surface irregularities due to corneal epithelial damage that are more associated with ADDE than DWDE. Those points that are useful for the differential diagnosis of DE will be reported elsewhere.

According to the findings in a multicenter study, in DWDE with SB, the subjective symptoms are very severe, which is compatible with what is observed in severe ADDE presenting AB [37]. Moreover, the symptom of severe pain in DWDE with SB can be objectively evaluated by PainVision^®^ (PS-2100; Osachi Co., Ltd., Nagano, Japan), an instrument used to assess pain severity [38]. In general, SB is not associated with corneal epithelial damage, and is thought to one of the BUPs in short-BUT type DE [16,18,19,20,21]. Therefore, SB is likely to be overlooked if the breakup pattern characteristic of SB is not diagnosed by the appropriate fluorescein staining of tears and verbal instruction given to the patient regarding the blink process [18,20]. If SB is not diagnosed properly, patients are not treated appropriately, which may lead to neuropathic pain due to a longstanding situation being left untreated [39]. Fortunately, in Japan, as well as in some other Asian countries, SB can be treated effectively using commercially available mucin secretagogues, such as diquafosol sodium or rebamipide eye-drops, which have both been demonstrated to enhance the expression of MUC16 [35,40]. Thus, and from this aspect also, it is important to diagnose SB appropriately and as early as possible.

It should be noted that this present study did have some limitations. One limitation was the lack of direct evidence concerning whether or not the characteristic profile of the disturbance value detected by videokeratography truly reflects SB. However, since only DE cases presenting reproducible SB were enrolled in this study, we postulate that the characteristic profile of DV must reflect SB. To verify this postulation, a system is required that can simultaneously observe videokeratography MR images and fluorescein-stained TF images. A second limitation is that we did not present any findings in this study as to whether or not our current VK method can effectively evaluate DE BUPs other than SB or RB, such as AB, LB, and DB. However, and as was mentioned above, considering the ability of the current DV to evaluate both TF stability and corneal epithelial damage, we assume that the detection of these BUPs must be possible, and we are now accumulating data to perform a comprehensive study to investigate the usefulness of DV for detecting other BUPs or DE subtypes. A third limitation is the possibility that in the real-world setting, DE with SB cannot be detected in eyes with a smaller palpebral width, or with eyelid diseases such as blepharoptosis, entropion, and ectropion, where DV can be miscalculated by eyelash shadows reflected upon the irregularities of the MR image. However, in this study, due to the fact that no cases with such diseases were enrolled, and that SB is likely to appear more commonly at the central cornea, negative effects such as those produced by eyelashes were neglected. However, in real-world situations, such effects might be given more consideration, and further study is necessary to clarify the impact of such effects on the findings. Apart from diagnostics of dry eye and contact lens compatibility, the videokeratography method coupled with novel image analysis and artificial intelligence algorithms will be useful for any type of diagnostics based on corneal topography measurements, e.g., planning of cataract or refractive surgery, and intraocular lens implantation.

## 5. Conclusions

The findings of the present study revealed that our new videokeratography method with a tracking system and a new disturbance value indicator can successfully detect an SB non-invasively and quantitatively, and showed that SB has a remarkably different DV profile from that of an RB. Considering that in comparison to the traditional method of using fluorescein, a non-invasive technique for the detection of SB allows more accurate and easier clinical implementation, the findings in this study should open the pathway for development of non-invasive TFOD via the assessment of BUPs and classification of DE subtype [17,18,19,20].

## Figures and Tables

**Table 1:** Demographic data of the Spot Break (SB) Group and Random break (RB) Group DE patients in the study.

	SB Group	RB Group	*p*
Patients (n)	21	23	
Female patients (n)	20	20	0.609
Mean age (years)	65.3 ± 8.50	66.9 ± 12.5	0.404

SB: spot break; RB: random break; DE: dry eye.

**Table 2:** Comparison of the OS abnormality and DV related parameters between the SB Group and RB Group.

	SB Group (n = 21)	RB Group (n = 23)	*p*
SG	1.476 ± 0.512	1.391 ± 0.583	0.485
CEDS	0.619 ± 0.865	0.261 ± 0.689	0.071
NIBUT	0 ± 0	7.156 ± 2.502	<0.001 *
FBUT	0 ± 0	3.836 ± 0.931	<0.001 *
TMR	0.188 ± 0.080	0.211 ± 0.082	0.346
DV(0 s)	77.2 ± 46.6	36.6 ± 21.8	<0.001 *
DV(2 s)	37.1 ± 29.9	27.5 ± 14.7	0.573
DV(5 s)	38.2 ± 30.1	28.6 ± 14.3	0.573
ΔDV(0 s–2 s)	−20.0 ± 14.4	−4.56 ± 8.78	<0.001 *
ΔDV(2 s–5 s)	0.38 ± 3.72	0.36 ± 2.55	0.725
ΣDV(0 s–2 s)	953 ± 697	570 ± 290	0.060
ΣDV(2 s–5 s)	1050 ± 774	810 ± 425	0.622

OS: ocular surface; DV: disturbance value; SB: spot break; RB: random break; SG: spread grade for tear film lipid layer; CEDS: corneal epithelial damage score; NIBUT: non-invasive breakup time. * *p*-value < 0.05.

**Table 3:** OS abnormality and disturbance value related parameters of representative Spot Break Group and Random Break Group cases.

	Case 1 (SB Group)	Case 2 (RB Group)
Patient age (years)	77	81
SG	1	2
CEDS	2	0
NIBUT	0	7.70
FBUT	0	4.67
TMR	0.095	0.136
DV(0 s)	108	6.48
DV(2 s)	18.0	7.26
DV(5 s)	31.3	12.2
ΔDV(0–2 s)	−45.0	0.393
ΔDV(2–5 s)	4.42	1.65
ΣDV(0–2 s)	844	156
ΣDV(2–5 s)	793	287

OS: ocular surface; DV: disturbance value; SB: spot break; RB: random break; SG: spread grade of the tear film lipid layer; CEDS: corneal epithelial damage score; NIBUT: non-invasive breakup time; FBUT: fluorescein breakup time; TMR: tear meniscus radius.

## Data Availability

The data that support the findings of this study are available from the corresponding author upon reasonable request.

## Supplementary Materials

The following supporting information can be downloaded at: https://www.mdpi.com/article/10.3390/diagnostics13020240/s1. Video S1: An image of the case in Figure 4 showing the video-recorded fluorescein-stained aqueous tear film dynamics immediately after eye opening, followed by keeping the eye open. Video S2: An image of the case in Figure 4 showing a video-recorded Meyer-ring image obtained via the current videokeratography system immediately after eye opening, followed by keeping the eye open. Video S3: An image of the case in Figure 5 showing the video-recorded fluorescein-stained aqueous tear film dynamics when the eye was kept open as long as 5 s. Video S4: An image of the case in Figure 5 showing a video-recorded Meyer-ring image obtained via the current videokeratography system when the eye was kept open as long as 5 s.

## Abbreviations

AB: area break; ADDE: aqueous deficient dry eye; ADES: Asia Dry Eye Society; BUP: breakup pattern; BUT: breakup time; BV: blur value; CEDS: corneal epithelial damage score; DB: dimple break; DE: dry eye; DEWS: Dry Eye Workshop; DV: disturbance value; DWDE: decreased wettability dry eye; FBUP: fluorescein breakup pattern; FBUT: fluorescein breakup time; IEDE: increased evaporation dry eye; LB: line break; MR: Meyer-ring; NIBUT: non-invasive breakup time; OS: ocular surface; RB: random break; SB: spot break; SD: standard deviation; SG: spread grade; TF: tear film; TFBU: tear film breakup; TFOD: tear film-oriented diagnosis; TFLL: tear film lipid layer; TFOT: tear film-oriented therapy; TM: tear meniscus; TMR: tear meniscus radius; VI: videointerferometry; VK: videokeratography; VM: videomeniscometry.
