# Supplementary material for: Successful Detection of the Characteristics of Tear Film Breakup Appearing Immediately after Eye Opening by Videokeratography with a Newly-Developed Indicator
Source: Diagnostics (Basel). 2023 Jan 9;13(2):240. doi: 10.3390/diagnostics13020240 (PMC9858532; doi:10.3390/diagnostics13020240)
Supplement: Supplementary file 1 [file diagnostics-13-00240-s001.zip › diagnostics-1949923-supplementary.pdf]

Supplementary Video S1

Video S1 is an image of the case in Figure 4 showing the video-recorded fluorescein-stained aqueous tear film dynamics immediately after eye opening, followed by keeping the eye open. Immediately after eye opening, spot break (SB) can be observed, some of which disappeared when the eye was kept open at around 2 s (s).

Supplementary Video S2

Video S2 is an image of the case in Figure 4 showing a video-recorded Meyer-ring image obtained via the current videokeratography system immediately after eye opening, followed by keeping the eye open. Immediately after eye opening, severe disturbance of the Meyer-ring can be observed, which attenuated to become stable at around 2 s. The time -dependent change of Meyer-ring immediately after eye opening and when the eye was kept open is thought to correspond to the dynamic tear film behavior in a case with SB.

Supplementary Video S3

Video S3 is an image of the case in Figure 5 showing the video-recorded fluorescein-stained aqueous tear film dynamics when the eye was kept open as long as 5 s. Aqueous tear film dynamics evaluated by fluorescein staining was very stable, which is compatible with a random break (RB) where fluorescein breakup occurs after stoppage of the upward movement of fluorescein (i.e., after the complete establishment of tear film).

Supplementary Video S4

Video S4 is an image of the case in Figure 5 showing a video-recorded Meyer-ring image obtained via the current videokeratography system when the eye was kept open as long as 5 s. The Meyer-ring was quite stable over time, excluding minor distortion of ring that appeared at 4.67 s after eye opening, which is thought to correspond to the dynamic tear film behavior in a case with RB.
